# Cost of Administration.—Hospitals for Consumption

**Published:** 1893-10-07

**Authors:** 


### The Institutional Workshop.

### HOSPITAL FINANCE.

### COST OF ADMINISTRATION.—HOSPITALS FOR CONSUMPTION.

In The Hospital for August 26th, pages 349-50, we dealt with the Metropolitan Consumption Hospitals, and gave the relative cost of administering each of the five hospitals included in this group. We showed that whereas a consumption hospital with 100 beds and upwards is administered for an average expenditure of less than 10 per cent. on management, those consumption hospitals which contain less than 100 beds expend considerably more than double this sum upon administration. We directed special attention to this point, and expressed a hope that we might receive some explanation of the causes which have produced results so unfavourable to the management of the smaller consumption hospitals as compared with that of the larger institutions in London. An attempt has been made to minimise the effect of those figures, which is worthy of a little detailed examination. We have always maintained that the bare statement that a bed cost so many pounds at one institution, and half as many pounds at another, does not necessarily, if at all, determine that the management of the one is infinitely superior to the other, or vice versâ. This contention is, no doubt, true, but it cannot in any way be held to justify excessive expenditure upon the administration of an institution because it happens to contain relatively few beds. For example, it is contended, in extenuation, that the average cost per bed occupied, for provisions, is higher at those consumption hospitals, namely, the Royal Chest and the North London, which contain less than 100 beds, than it is at the Brompton and Victoria Park Hospitals, which contain 321 and 164 beds respectively. The fallacy of such a contention will become apparent when we point out that had the two smaller hospitals expended no more per occupied bed for provisions than the two larger institutions, the percentage of their cost of management to that of maintenance would have been considerably higher than it is under present circumstances.

### The Medical Staff and Expenditure.

It is urged that in the expenditure upon all such matters as food and drugs, the lay committee have very little, if any, control, the dietary table for the patients being prepared by a committee of the medical staff; and that all alcohol and drugs are, of course, given under their directions. The latter contention is, no doubt, true, but the managers of the Edinburgh Royal Infirmary have proved the possibility of instituting a system, which by bringing to the notice of each member of the medical staff, once a month, the actual money value of the dressings, drugs, alcohol, &c., consumed by the patients for which he is individually responsible, and by instituting a comparison between the outlay upon the patients under the charge of each physician and surgeon, a considerable reduction in expenditure is obtainable. Not only is the cost of each patient thereby reduced, but something like uniformity of practice can be maintained, with great advantage to all concerned. We have no doubt, therefore, that if the respective committees take steps to acquaint the members of the medical staff of the Royal Chest and the North London Hospitals with the fact that the average cost per bed occupied, upon the items referred to, is larger at these institutions than it is at the Brompton and Victoria Park Hospitals, an inquiry will be instituted which cannot fail to be attended by important results. We are confident that every member of the medical staff will be found most anxious to co-operate with the lay committee to secure the utmost economy, combined with efficiency, throughout each institution. We commend the system enforced at the Royal Edinburgh Infimary to the attention of the committees of the Royal Chest and the North London Hospitals, full details of which will be found in the third volume of "Hospitals and Asylums of the World."

### Money Spent on Administration.

It has been erroneously stated that this constitutes our *bête noir*. This is a palpable absurdity, as we have made it abundantly clear in the series of articles we have published during the last few weeks, that each class of hospitals is entitled to spend a certain maximum sum upon administration. We have even gone so far as to point out that, in our view, too little money was in certain cases spent under this head. We have further emphasized the importance of remembering that the circumstances of each hospital, and even of particular hospitals in each group, justify a relatively large or moderate expenditure, according to the special circumstances of each institution. For example, in the case of Charing Cross and King's College Hospitals, where the reliable income is unfortunately small, and where the sum to be raised annually in benefactions is very large, the expenditure upon administration must necessarily be much larger than in the case of St. George's Hospital, where the income from invested funds and annual subscriptions is happily large. Any one who wishes to give wisely to the hospitals, and we take it that no man of business who examines the tables we have published will fail to grasp these differences, ought to make due allowance for them in apportioning the amount of his subscription to a particular hospital. What the defenders of certain special hospitals fail to grasp is that there must be a maximum sum, beyond which expenditure upon management must not extend. It is perfectly clear that a man of business who had money to give to charities would prefer to send it to an institution where he knows that 90 per cent. of his gift will be spent in carrying out the benevolent objects of the institution. Otherwise serious retrogression in matters of hospital finance must ensue, and the old bad days will return, when the promoters of special hospitals may again contend that it does not matter how much money they spend in raising an income, provided they secure sufficient funds to pay their way. We have been most careful to avoid unjust or harsh criticism in dealing with every group of hospitals, but it is essential that the giving public should be made to understand that any hospital which expends more than 20 per cent. of its income upon management, is one which ought properly to be regarded as suspect, unless or until its managers show cause for this exaggerated expenditure.

Two Points of Importance.

The cost of administration is but one of several methods of testing the efficiency of the management of a particular institution. This is indirectly shown by the circumstance that the apologists for the large expenditure upon management at the Royal Chest and North London Hospitals adduce as a point in their favour the fact that they spend more for provisions than the larger hospitals in the same class. Not only is this not maintainable, but it is even conceivable that the management of a particular institution might be so bad in every department as to conceal the enormous expenditure upon management by sanctioning the most extravagant expenditure upon maintenance. Every hospital committee must make up its mind to fix the maximum of expenditure upon management, and at the same time to keep a strict eye upon every other item of expenditure. It is a mistake to suppose that lavish expenditure means efficiency in administration. Our experience, and it extends to all classes of hospitals, confirms the view that there is a limit of cost which should never be exceeded, and which in practice seldom or never is exceeded, in the best administered hospitals. It must further be patent that heavy administration expenses, that is an expenditure upon management larger than the maximum which the circumstances and difficulties of each particular group warrant, and which the lay committee, being men of business, can readily fix, does mean bad management, and should result in a diminished subscription list. We are confident that certain special hospitals spend a great deal too much money upon advertising. There is no item of expenditure which requires greater judgment and care than money spent upon advertisements. Indeed, we are inclined to believe that beyond a certain definite and limited sum, waste rather than benefit follows outlay under this head.

What the Committees Might Do!

It is noticeable that our critics do not attempt to question the accuracy of the figures we have published. They have misconceived the object of these articles, which is to secure an adequate supply of hospital beds of every kind, for the comfort and succour of the suffering poor. Consumption may be regarded as the curse of our climate; it spares neither prince nor peasant; it is met with in the homes of the wealthy, and in the cottages of the poor, and so all classes, and each class for itself, is personally interested in securing an adequate number of hospital beds in our consumption hospitals, so that science may have the fullest opportunity of discovering the best methods of treatment, and each of us may do our best to alleviate a form of human misery which appeals, by the very nature of its insidious and fatal progress, to the best instincts of every healthy man and woman in our midst. We have shown on several occasions that London, at the present time, urgently needs many hundreds more beds for the reception of cases of phthisis. We have further shown that consumption hospitals, with less than 100 beds, as at present conducted, entail too much expenditure upon management. The North London Hospital has an ample site upon which a building, capable of containing 200 more beds, might readily be erected. The Royal Chest Hospital contains 80 beds, of which only half have heretofore been occupied, on an average. Will not the committees of these two institutions either separately or jointly take steps to awaken the public to the urgent necessity of providing additional beds within their institutions for the reception of patients? If they do so successfully, the cost of management will speedily assume moderate proportions. The emergency is a special one, calling for strenuous efforts, and the institution of special measures adequate to meet it. In such circumstances the Committees would be justified in voting some hundreds of pounds to be devoted entirely to the purpose of raising the necessary money to enable the vacant beds to be filled at the Royal Chest Hospital, and further accommodation to be provided for the North London Hospital also.

Of Importance to All.

Once more we urge that this question of providing an adequate number of beds for consumption cases is a country quite as much, if not more, than it is a metropolitan one, because so many patients in the consumption hospitals are at present sent from the country. Hundreds of thousands of pounds have been spent of recent years upon technical education. Is it too much to urge the increasing number of wealthy people who desire to give of their abundance substantial sums for proved objects of public utility, to turn their attention to this pressing question without delay or hesitation? One hundred thousand pounds is not a large sum, and yet it would do much to enable the managers of our consumption hospitals to grapple with the ever-increasing demands of suffering humanity, which have, alas! at present, in so many cases to be disregarded. Let those of us at any rate who have a knowledge of the facts, and who yield to none in our desire to see the good work adequately extended, decline to waste time in arguing points of detail. Let us, rather, combine to prevent the further prolongation of a state of affairs which causes an ever-increasing amount of human misery, by providing comforts and treatment which must, under Providence, tend to prolong the lives of thousands of men and women.

